# Comparative Proteomics Analysis Reveals the Reversal Effect of Cryptotanshinone on Gefitinib-Resistant Cells in Epidermal Growth Factor Receptor-Mutant Lung Cancer

**DOI:** 10.3389/fphar.2022.837055

**Published:** 2022-03-10

**Authors:** Peiheng Cai, Gaofan Sheng, Shiqin Jiang, Daifei Wang, Zhongxiang Zhao, Min Huang, Jing Jin

**Affiliations:** ^1^ School of Pharmaceutical Sciences, Sun Yat-sen University, Guangzhou, China; ^2^ School of Chinese Materia Medica, Guangzhou University of Chinese Medicine, Guangzhou, China

**Keywords:** cryptotanshinone, gefitinib, resistance, proteomics, nonsmall cell lung cancer

## Abstract

Cryptotanshinone (CTS) is a lipophilic constituent of *Salvia miltiorrhiza*, with a broad-spectrum anticancer activity. We have observed that CTS enhances the efficacy of gefitinib in human lung cancer H1975 cells, yet little is known about its molecular mechanism. To explore how CTS enhances H1975 cell sensitivity to gefitinib, we figured out differential proteins of H1975 cells treated by gefitinib alone or in combination with CTS using label-free liquid chromatography-tandem mass spectrometry (LC-MS/MS). Gene Ontology (GO), Kyoto Encyclopedia of Genes and Genomes (KEGG), and protein–protein interaction (PPI) bioinformatic analyses of the differential proteins were performed. CTS enhanced H1975 cell sensitivity to gefitinib *in vitro* and *in vivo*, with 115 and 128 differential proteins identified, respectively. GO enrichment, KEGG analysis, and PPI network comprehensively demonstrated that CTS mainly impacted the redox process and fatty acid metabolism in H1975 cells. Moreover, three differential proteins, namely, catalase (CAT), heme oxygenase 1 (HMOX1), and stearoyl-CoA desaturase (SCD) were validated by RT-qPCR and Western blot. In conclusion, we used a proteomic method to study the mechanism of CTS enhancing gefitinib sensitivity in H1975 cells. Our finding reveals the potential protein targets of CTS in overcoming gefitinib resistance, which may be therapeutical targets in lung cancer.

## Introduction

Lung cancer is one of the most malignant tumors, with high morbidity and mortality over the world. About 80% of lung cancers are non-small cell lung cancers (NSCLCs), and epidermal growth factor receptor (EGFR) gene mutations are considered to prompt NSCLC development (Sharma et al., 2007). Although EGFR-tyrosine kinase inhibitors (TKIs) have shown good clinical efficacy, therapy resistance inevitably occurs. With long-term use of first-line EGFR-TKIs, most patients would develop acquired resistance after a median of 12 months (Nagano et al., 2018). Mechanisms of resistance to EGFR-TKIs are complicated, including T790M secondary mutation, activation of alternative or downstream pathways, histological transformation, etc., (Yu et al., 2014). Hence overcoming the resistance to EGFR-TKIs has become a hotspot of research.

Cryptotanshinone (CTS), a lipid-soluble compound extracted from the roots of traditional Chinese medicine *Salvia miltiorrhiza*, is mainly used to treat cardiovascular and inflammatory diseases (Li et al., 2021). Recent studies show that CTS also has antitumor properties, mainly by inducing apoptosis, inhibiting cancer cell proliferation, metastasis and invasion, inhibiting angiogenesis, and drug efflux (Ashrafizadeh et al., 2021). Although CTS has been found to reverse chemoresistance by multiple mechanisms, however, the mechanism of CTS in enhancing gefitinib sensitivity is still unclear.

Differential proteomics, also known as comparative proteomics, studies the changes in proteome in different physiological or pathological states between samples in order to find key differential proteins as markers for qualitative and functional analysis (Yang et al., 2019). Over the years, many techniques have been developed for protein separation, digestion, enrichment, identification, as well as absolute and relative quantification (Megger et al., 2013). One quantitative method is labeling-based quantification, represented by stable isotope labeling by amino acids in cell culture (SILAC) and isobaric tags for relative and absolute quantification (iTRAQ). The other is label-free quantification utilizing LC-MS/MS for relative quantification, which has a large dynamic range and high proteome coverage (Li et al., 2012). Differential proteomics has been widely used in the mechanism study of traditional Chinese medicines at the protein level (Yang et al., 2019). Comparative proteomic studies of anticancer phytochemicals have found plentiful targets, such as cyclins, cytoskeleton proteins, and metabolic enzymes (Wong et al., 2016), either confirming known cancer biomarkers or uncovering new ones. For example, a study employing two-dimensional difference gel electrophoresis with mass spectrometry to investigate the effect of honokiol in human thyroid cancer cells showed that honokiol regulates proteins involving cytoskeleton, protein folding, transcription control, and glycolysis (Chou et al., 2018). Another study using the iTRAQ method to study the mechanism of triptolide in A549 cells found candidate proteins involved in the PARP1/AIF pathway, nuclear Akt signaling, and metastasis processes (Li et al., 2018).

This article reports that CTS increases the sensitivity of gefitinib-resistant H1975 cells to gefitinib, a first-line EGFR-TKI. To explore its mechanism, label-free proteomics is used to identify the differential proteins under gefitinib treatment alone or in combination with CTS. The expression levels of three selected differential proteins, CAT, HMOX1, and SCD were verified. This study reveals the potential targets of CTS in H1975 cells, providing evidence of CTS as a gefitinib sensitizer to overcome resistance in clinical cancer therapy.

## Materials and methods

### Chemicals and reagents

Ammonium bicarbonate (NH_4_HCO_3_), ethanol, formic acid (FA), chloroform (CHCl_3_), and isopropanol with analytical purity were purchased from Tianjin ZhiYuan Reagent Co., Ltd., (Tianjin, China). Acetone, acetonitrile (ACN), urea, dithiothreitol (DTT), and trifluoroacetic acid (TFA), with purity higher than 99.0%, were purchased from Shanghai Aladdin Biochemical Technology Co., Ltd., (Shanghai, China). DMSO and iodoacetamide (IAM) with purity higher than 99% was purchased from Sigma-Aldrich Trading Co. Ltd., (MO, United States). Gefitinib was purchased from Yuanye Bio-Technology Co., Ltd., (Shanghai, China), and CTS was purchased from Winherb (Shanghai, China).

### Cell culture and viability assay

Human lung cancer H1975 cells were obtained from the National Collection of Authenticated Cell Cultures (Shanghai, China). H1975 cells were maintained in RPMI-1640 medium (Corning, United States) containing 10% FBS (Gibco, United States) and 1% penicillin/streptomycin (Cellgro, United States) at 37°C and 5% CO_2_. Gefitinib (4.47 mg) and CTS (2.96 mg) were dissolved, respectively, in 100 μl of DMSO as the stock solutions, stored at −20°C, and diluted with culture medium before use. Before treatment, cells were seeded in 96-well plates (Corning, United States) at a density of 5 × 10^3^ cells per well and allowed to attach overnight. Cells were exposed to gefitinib (10, 20, and 40 μM), CTS (2.5 and 5 μM), and combinations of them, respectively, for 72 h. Cell viability was tested by CCK-8 (Dojindo, Japan) according to the manufacturer’s instructions. Absorbance was measured at 450 nm by a microplate reader (THERMO Electron, United States). Cell survival rate was calculated. Each group has four replicates.

### Animal model and treatment

Animal experiments were performed with the approval of the Animal Ethical and Welfare Committee of Sun Yat-sen University. SPF male BALB/c mice weighing 18–22 g at 3–4 months were purchased from the Beijing Vital River Laboratory Animal Technology Co. Ltd. All mice were kept in a specific pathogen-free animal room at 20°C–25°C and 40–70% humidity with a 12 h light–dark cycle, provided with water and feed *ad libitum*. H1975 cells were suspended in PBS and injected subcutaneously to the left armpit of the mice (5 × 10^6^ cells/0.1 ml per mouse). The formula width^2^ × length2 was used to measure tumor size. Once the tumor size reached 50–100 mm^3^ (within 5–7 days), the mice were randomly assigned into four groups (n = 10 mice/group) for daily drug administration: 1) vehicle control group (1% Tween-80 ig. and corn oil ip.); 2) Gef group (50 mg/kg Gef ig. and corn oil ip.); 3) CTS group (1% Tween-80 ig. and 20 mg/kg CTS ip.); and 4) Gef + CTS group (50 mg/kg Gef ig. and 20 mg/kg CTS ip.). After 17 days of administration, mice were sacrificed. Tumor tissues were weighed and stored at −80°C.

### Sample preparation for proteome

#### Protein extraction

Treated cells were washed with PBS (Gibco, USA) and lysed with RIPA containing 1% PMSF (both from Beyotime, China) on ice for 30 min. Cell lysate was scraped, collected, and sonicated with 35% amplitude on ice for 10–15 s. After centrifugation at 12,000 × *g*, 4°C for 20 min in a centrifuge (Eppendorf, Germany), the supernatant was obtained as cellular protein solution. For tissue sample, 100 mg of tissue in 1 ml of RIPA containing 1% PMSF was homogenated four times under 5,000 rpm for 8 s with a 10 s interval by a homogenator (Birtin, France). After centrifugation at 12,000 × *g*, 4°C for 20 min, the supernatant was obtained as tissue protein solution. Protein concentrations were measured with a BCA Protein Assay Kit (Thermo, United States).

#### Acetone Precipitation and Enzymatic Hydrolysis

The protein solution (300 μg of protein diluted with 50 mM NH_4_HCO_3_ to 100 μl) was added with 400 μl of cold acetone, mixed, and precipitated overnight at 4°C. After centrifugation at 15,000 × *g*, 4°C for 30 min, the supernatant was discarded, and the precipitate was washed with cold acetone, 70% ethanol, and acetone in sequence, 500 μl for each. The sample was centrifuged at 15,000 × *g*, 4°C for 30 min after each wash, and the supernatant was discarded. After evaporated to dry in a fume hood, the final precipitate was resuspended with 50 μl of 8 M UA buffer (in 0.1 M Tris/HCl, pH 8.5), shaken at room temperature for 2 h until the precipitate was completely dissolved. A 2 μl diluted DTT solution was added (DTT final concentration = 2 mM), mixed, and the sample was placed in a 30°C water bath for 1.5 h. Then 13 μl of diluted IAM solution was added (IAM final concentration = 10 mM), mixed, and the sample was placed in darkness for 40 min. Then it was diluted with 50 mM NH_4_HCO_3_ to 600 μl (urea final concentration= 0.7 mol/L), added with 20 μl of 0.25 μg/μl Trypsin (Promega, United States) with trypsin/protein ratio 1:60 (w/w), mixed, and hydrolyzed overnight at 37°C. Then 10% TFA was used to stop the reaction by acidifying the solution to a final concentration of 0.4%.

#### C18 Stage Tip Peptide Desalting

To activate C18 stage tip, 200 μl of methanol was added, centrifuged at 1,200 × *g* for 10 min, and the effluent was discarded, and repeated three times. To equilibrate tip, 200 μl of 80% ACN/0.1% FA was added, centrifuged at 4,000 × *g* for 4 min, and the effluent was discarded, and repeated three times. Then 200 μl of 0.1% TFA was added to tip, centrifuged at 6,000 × *g* for 4 min, the effluent was discarded, and repeated three times. A 5 μg digested protein sample was dissolved in 200 μl of 0.1% TFA, and the sample was loaded twice, centrifuged at 2,000 × *g* for 12 min, and the effluent was discarded. For salt elution, 200 μl of 0.1% TFA was added to tip, centrifuged at 6,000 × *g* for 4 min, and the effluent was discarded. For TFA elution, 200 μl of 0.1% FA was added to tip, centrifuged at 6,000 × *g* for 4 min, and the effluent was discarded, and repeated twice. Then 180 μl of 80% ACN/0.1% FA was added to tip, centrifuged at 2,000 × *g* for 4 min, and repeated twice. The effluent was collected and vacuum dried overnight. The dried sample was redissolved with 10 μl of 0.1% FA and centrifuged at 16,000 × *g* for 10 min. The supernatant was transferred to the sample bottle.

### Label-free liquid chromatography-tandem mass spectrometry analysis

A C18 column (75 μm × 150 mm, NanoViper C18, 2 μm, 100 Å; Thermo, PA, United States) was used to separate peptide samples in 0.1% formic acid in the Nano LC-Q Exactive Plus mass spectrometer (Thermo, United States). Eluent A was 0.1% formic acid aqueous solution, and eluent B was 0.1% formic acid 80% acetonitrile solution. The elution gradient is set as follows: 0–95 min, 3%→32% (B); 95–105 min, 32%→100% (B); 105–120 min, 100% (B), the total running time is 120 min at a flow rate of 300 nl/min. All MS and MS2 spectra were collected, where the 10 strongest ions were fragmented by collision-induced dissociation. A full mass scan in the range of 355–1,700 m/z was obtained, with a mass resolution of 70,000. The LC-MS/MS data were analyzed by searching the UniProt Human database (https://www.uniprot.org/proteomes/UP000005640) downloaded on December 7, 2017 using Thermo Proteome Discoverer 2.2. Parameters were set, and some modifications were made as described: Fixed modification: ureido modification; dynamic modification: oxidation modification; enzyme: trypsin; precursor mass tolerance: 20 ppm, maximum; missing cut sites: 2; fragment quality tolerance: 0.02 Da; target false discovery rate (FDR) (strict): 0.01; and target FDR (loose): 0.05. Verification was based on the Q value. Result was exported from Thermo Proteome Discoverer 2.2 to Excel for analysis. Proteins meeting all the criteria (*p* < 0.05, high protein FDR confidence, and abundance ratio > 1.5 or <0.67) were recognized as differential proteins.

### Bioinformatics analysis

GO enrichment analysis of differential proteins was performed using DAVID 6.8 (https://david.ncifcrf.gov/). Pathway analysis was performed on KEGG platform (http://kobas.cbi.pku.edu.cn/index.php). PPI analysis was performed using STRING 11.0 (https://string-db.org/cgi/input.pl).

### Western blot analysis

Protein samples were prepared as described in protein extraction and denatured by loading buffer. Protein, 25 μg per sample, was electrophoresed on SDS-PAGE gels (10%–12%) and blotted onto PVDF membranes (Amersham Hybond, United States). After blocking with 5% milk, the membranes were incubated at 4°C overnight with the following primary antibodies: *β*-actin (Cell Signaling, United States), catalase (CAT, Sangon, China), heme oxygenase 1 (HMOX1, Santa Cruz, United States), and stearoyl-CoA desaturase (SCD, Sangon, China). Anti-rabbit secondary antibodies (Cell Signaling, United States) were used. Target protein expressions were detected by chemiluminescence and quantified using ImageJ.

### Quantitative RT-PCR

Treated cells in 12-well plates were washed with PBS, added with 500 μl of Trizol (Takara, Japan). After 15 min of incubation at 4°C with shaking, cell lysate was obtained and added with 500 μl of CHCl_3_. For tissue sample, 50 mg of tissue in 500 μl of Trizol was homogenated four times under 5,000 rpm for 8 s with a 10 s interval in a homogenator. A 400 μl supernatant was transferred and added with 80 μl of CHCl_3_. Cell lysate or tissue supernatant added with CHCl_3_ was vortexed for 15 min, let stay for 3 min, and then centrifuged at 12,000 × *g*, 4°C for 15 min; 100 μl of supernatant was transferred and added with 200 μl of isopropanol, and gently shaken upside down 15 times. After staying for 10 min, the sample was centrifuged at 4°C and 12,000 × *g* for 10 min, and the supernatant was discarded as much as possible. Precipitate was upside down washed with 400 μl of 75% ethanol in diethyl pyrocarbonate (DEPC) water solution. The sample was centrifuged at 12,000 × *g*, 4°C for 8 min. The supernatant was removed as much as possible, and the precipitate was dried in a fume hood. Dried precipitate was dissolved in 20 μl of DEPC water, and the RNA concentration was measured by Nanodrop 2000 (Thermo Fisher, United States). Genome DNA was removed, and reverse transcription was performed using Prime Script RT reagent kit (Takara, Japan), and RT-qPCR was performed using Takara SYBR ^®^ Premix Ex Taq™ II (Takara, Japan) in a 7500 Real Time PCR System (Applied Biosystems, United States). Sequences of primers (Sangon, China) were as follows: *β*-actin (forward: CAT​GTA​CGT​TGC​TAT​CCA​GGC, reverse: CTC​CTT​AAT​GTC​ACG​CAC​GAT), CAT (forward: TGG​AGC​TGG​TAA​CCC​AGT​AGG, reverse: CCT​TTG​CCT​TGG​AGT​ATT​TGG​TA), HMOX1 (forward: AAG​ACT​GCG​TTC​CTG​CTC​AAC, reverse: AAA​GCC​CTA​CAG​CAA​CTG​TCG), and SCD (forward: TCT​AGC​TCC​TAT​ACC​ACC​ACC​A, reverse: TCG​TCT​CCA​ACT​TAT​CTC​CTC​C).

### Statistical analysis

All experiments were repeated three times. All data were in mean ± SD. GraphPad Prism 7 was used for data analysis and drawing. Results were considered statistically significant when *p* < 0.05.

## Results

### Cryptotanshinone inhibits H1975 cell proliferation and sensitizes H1975 cell to gefitinib

**FIGURE 1:**
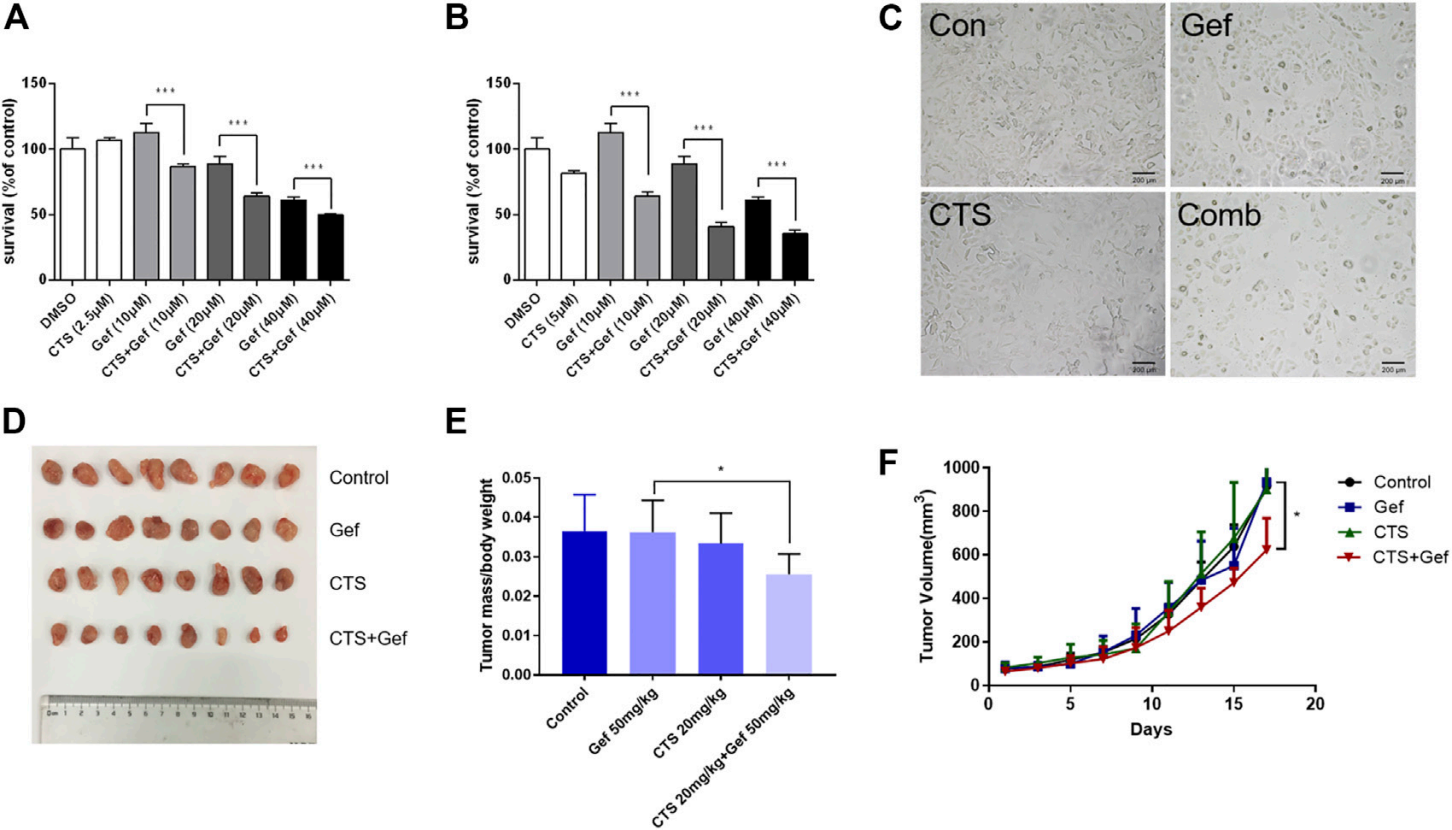
CTS increases the sensitivity to gefitinib in H1975 cells and xenografts in nude mice. **(A,B)** H1975 cells were treated with different concentrations of gefitinib (0, 10, 20, and 40 μM) alone or in combination with 2.5 μM **(A)** or 5 μM **(B)** CTS for 72 h. The cell viability was determined by CCK-8 (*n* = 5). Data are represented as the means ± SD, and significant differences are indicated as *p < 0.05, **p < 0.01, and ***p < 0.001. **(C)** H1975 cells were treated with 0.1% DMSO, 20 μM Gef, 5 μM CTS, and 20 μM Gef with 5 μM CTS, respectively, and cell morphology was imaged. **(D–F)** Tumor-bearing BALB/c nude mice were assigned into four groups (*n* = 8 per group) and treated with vehicle, Gef (50 mg/kg), CTS (20 mg/kg), and a combination of CTS and Gef for 17 days. Tumors in each treatment group were photographed **(D),** and tumor weights were measured **(E)**. Tumor volumes were measured every 2 days **(F)**.

To investigate the effect of cryptotanshinone (CTS) on chemosensitivity of human lung cancer, the gefitinib-resistant H1975 cells were treated with different concentrations of gefitinib alone or together with CTS. After 72 h of treatment, cell viability reduced significantly under combinatorial treatment of CTS and gefitinib (CTS + Gef) than gefitinib (Gef) alone (Figures 1A, B). In addition, cell number reduction was confirmed by microscopic observation (Figure 1C). The drug interaction coefficients (CDI) were below 0.8 (<1), indicating that CTS enhanced the sensitivity of H1975 cells to Gef.

To examine the sensitizing effect of CTS *in vivo*, BALB/c mice with H1975 subcutaneous xenograft were established. Although no significant tumor-inhibitory effect could be observed under CTS or Gef treatment, combination of CTS and Gef markedly inhibited tumor growth (Figures 1D–F), with 34.59% inhibitory efficiency compared with the Gef group. The Q value is 5.32 (>1.15), indicating a synergistic effect of CTS plus Gef.

### Identification of differential proteins under gefitinib and cryptotanshinone combination treatment versus gefitinib monotherapy

**FIGURE 2:**
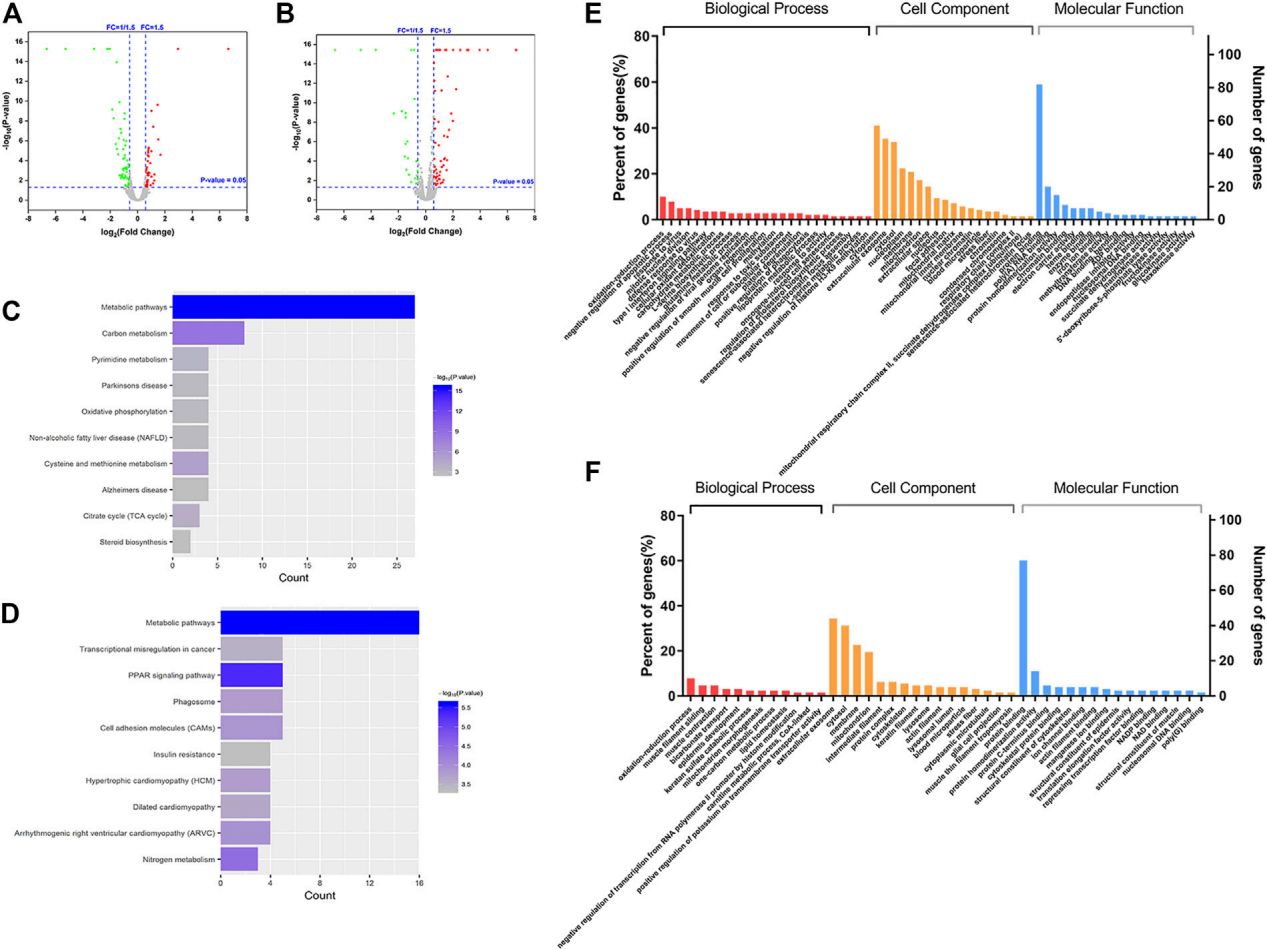
Differential proteins between the Gef group and the CTS + Gef group displayed with volcano plots, Gene Ontology (GO) functional annotation, and Kyoto Encyclopedia of Genes and Genomes (KEGG) pathway analysis. Volcano plot analysis of differential proteins *in vitro* **(A)** and *in vivo* **(B)**. KEGG pathway analysis of differential proteins *in vitro* **(C)** and *in vivo* **(D)**. GO functional annotation of differential proteins *in vitro* **(E)** and *in vivo* **(F)**.

In order to identify the differential proteins, cell and tissue samples from the Gef group and the CTS + Gef group were quantitatively analyzed with Nano LC-Q Exactive Plus. Under the criteria of fold change >1.5 or <0.67, totally 115 and 128 differential proteins were detected in cell and tissue samples, respectively (Figures 2A, B). There were 58 proteins downregulated and 57 upregulated in cell samples; there were 43 proteins downregulated and 85 upregulated in tissue samples, as listed in the Supplementary Material.

### Gene Ontology analysis

The differential proteins were annotated with Gene Ontology (GO) functions, with their *p*-value scores. The differential proteins in cell samples between the Gef group and the CTS + Gef group were located primarily in the cytoplasm and extracellular matrix. The main biological activities these proteins were involved in were redox process, blood coagulation process, protein binding, protein homodimerization, and RNA binding (Figure 2E). Differential proteins in tissue samples were located primarily in the extracellular matrix and cytoplasm. They were mainly involved in the redox process, muscle contraction, protein binding, and protein homodimerization (Figure 2F). Therefore, the differential proteins between the Gef group and the combination group had the most enriched GO terms in redox process and protein binding.

### Kyoto Encyclopedia of Genes and Genomes pathway analysis

The pathways related to differential proteins were revealed by KEGG pathway analysis. In cell samples, the differential proteins were closely related to metabolic pathways, carbon metabolism, pyrimidine metabolism, oxidative phosphorylation, cysteine and methionine metabolism, and TCA cycle (Figure 2C). The differential proteins in tissue samples were closely associated with metabolic pathways, transcriptional misregulation in cancer, PPAR signaling pathways, phagosome, cell adhesion molecules, and nitrogen metabolism (Figure 2D). It was deduced that CTS exerts its effect through metabolic pathway change.

### Protein–protein interaction network analysis

**FIGURE 3:**
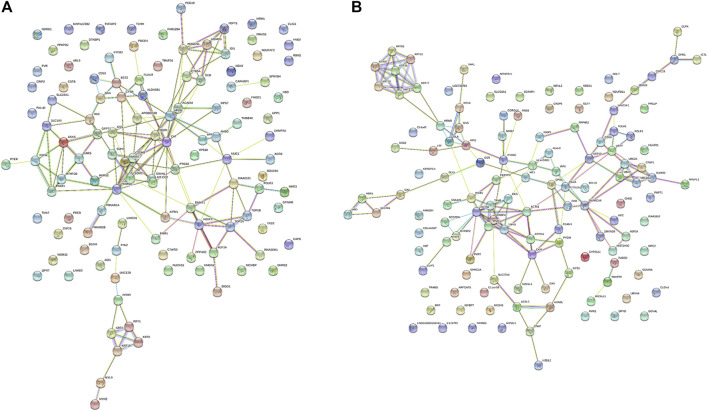
The protein–protein interaction (PPI) network of differential proteins between the Gef group and the CTS + Gef group. The PPI network of differential proteins in H1975 cells **(A)** and tumor tissues **(B)**.

**FIGURE 4:**
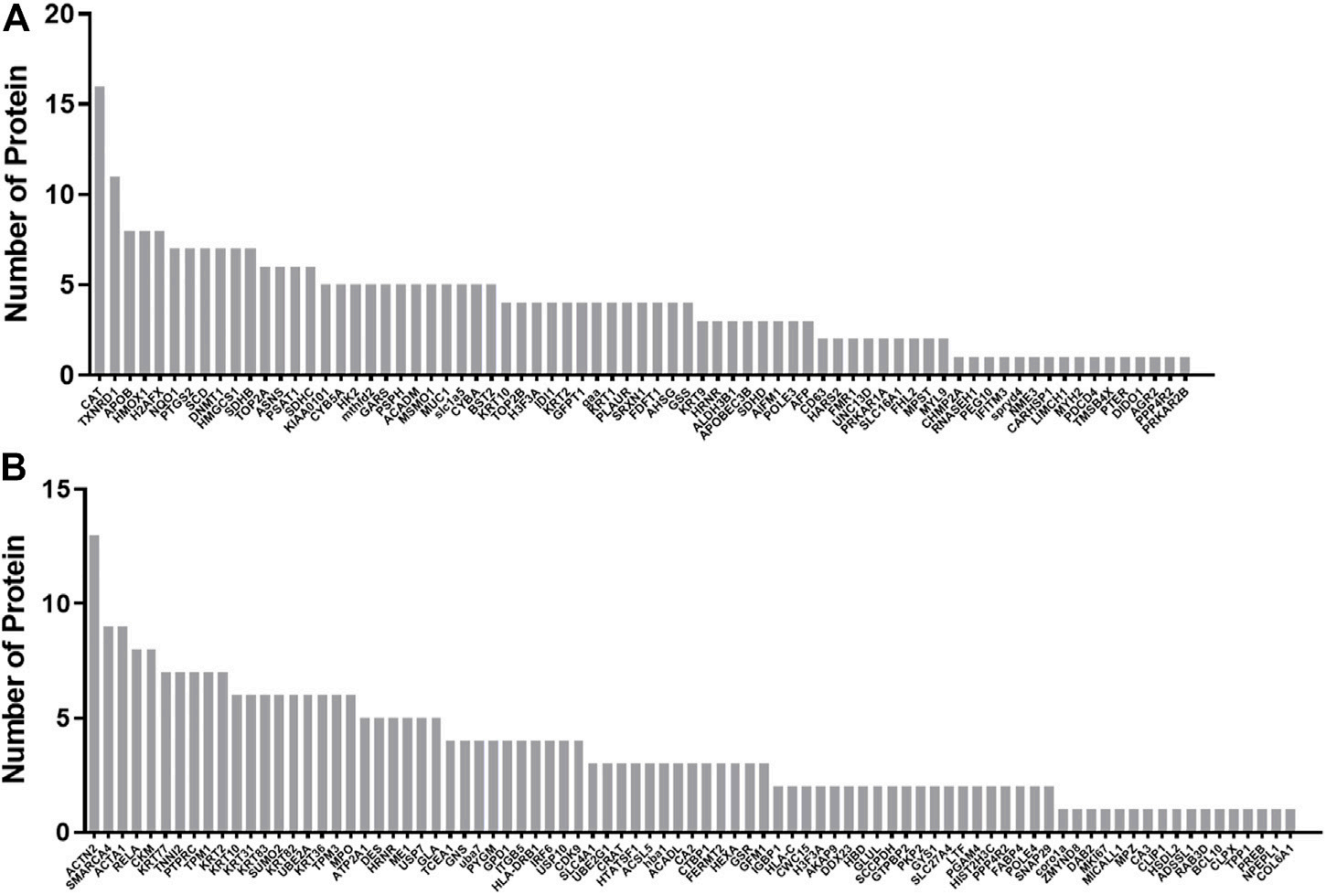
Statistics of interactions with differential proteins from the PPI network. Statistics of interactions with differential proteins in H1975 cells **(A)** and tumor tissues **(B)** from the PPI network.

The protein–protein interaction network among the differential proteins are shown (Figures 3A,B), with the number of interacting proteins counted (Figures 4A,B). In cell samples, 115 differential proteins constituted a PPI network, which displayed a central cluster with CAT as the core, surrounded by TXNRD1 (thioredoxin reductase 1), HMOX1, NQO1 NAD(P)H (quinone oxidoreductase 1), SCD, and other proteins related to oxidative stress and fatty acid metabolism (Figure 3A). In tissue samples, 128 differential proteins also constituted a PPI network, at the core of which was FABP4 (fatty acid-binding protein 4) linked with other proteins related to carbohydrate metabolism and fatty acid metabolism (Figure 3B). This comprehensive analysis implied that CTS may enhance Gef sensitivity by impacting the oxidative stress pathway and fatty acid metabolism in cancer cells.

### RT-qPCR and Western blot validation of differential proteins

**FIGURE 5:**
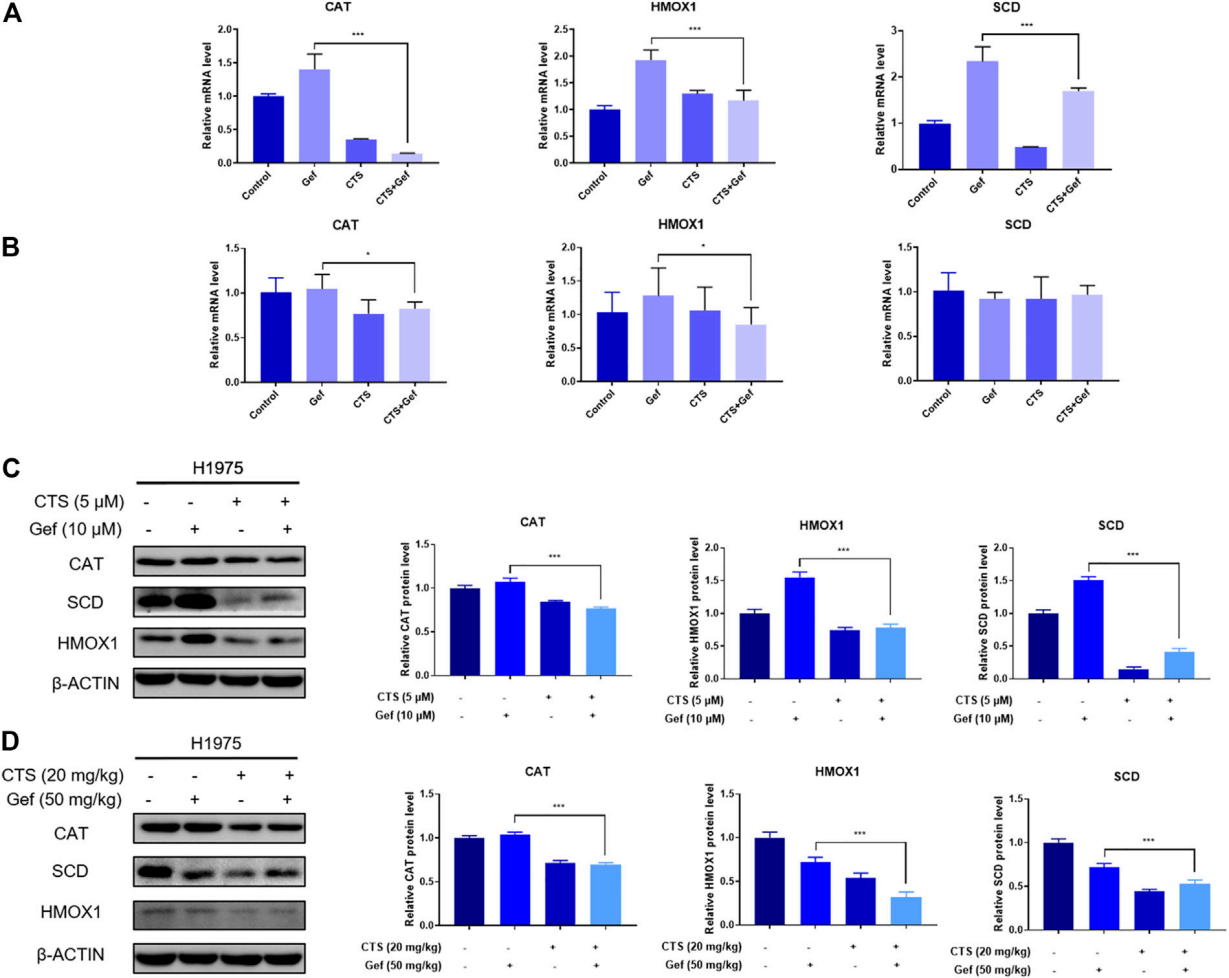
CTS or/and Gef treatment regulated the transcription and expression levels of the differential proteins. **(A,C)** The mRNA **(A)** and protein **(C)** levels of CAT, HMOX1, and SCD in H1975 cells under combination of CTS (5 μM) and Gef (10 μM) compared with Gef (10 μM) for 72 h. **(B,D)** The mRNA **(B)** and protein **(D)** levels of CAT, HMOX1, and SCD in tumor tissues under combination of CTS (20 mg/kg, ip) and Gef (50 mg/kg, ig) compared with Gef (50 mg/kg, ig) for 17 days. Data are represented as the means ± SD (*n* = 3), and significant differences are indicated as *p < 0.05, **p < 0.01, and ***p < 0.001.

The mRNA levels of differential proteins were examined by RT-qPCR. The *in vitro* mRNA levels of CAT, HMOX1, and SCD were markedly increased in the Gef group than those in the control, but reduced after combination treatment compared to the Gef group (Figure 5A). The *in vivo* mRNA levels of CAT and HMOX1 were significantly reduced with combination treatment than those with Gef, but no significant difference was found in the mRNA level of SCD (Figure 5B). Expressions of the differential proteins were verified by Western blot. As expected, the levels of CAT, HMOX1, and SCD in the combination group were significantly lower than those in the Gef group, both *in vitro* and *in vivo* (Figures 5C, D). Therefore, combination treatment of CTS and Gef significantly suppressed the expression of CAT, HMOX1, and SCD compared with Gef treatment.

## Discussion

The use of traditional herbal medicines in anticancer therapy is a promising strategy to overcome resistance. The active constituent from traditional Chinese medicine *Salvia miltiorrhiza*, CTS, is found with antineoplastic activity and promotes the efficacy of many anticancer drugs (Fu et al., 2020). In this study, we discovered that CTS enhances the sensitivity of H1975 cells to gefitinib. To identify the molecular targets of CTS in H1975, we employed label-free proteomics and found the differential proteins in H1975 cells under gefitinib monotherapy or gefitinib in combination with CTS. Differential proteins *in vitro* and *in vivo* were further grouped into different GO terms and KEGG pathways. Results showed that redox process and protein binding are the most enriched GO terms, and metabolic pathway is the most relevant pathway. In addition, PPI analysis further revealed the interaction network of these differential proteins. Collectively, our study provides a landscape of proteins and their related pathways regulated by CTS in H1975 cells treated with gefitinib.

Core proteins in the PPI network, namely, CAT, HMOX1, and SCD, were chosen and validated by RT-qPCR and Western blot. These proteins exhibit an increase under gefitinib treatment, but show a decrease after a combination of CTS with gefitinib. Thus, we inferred that these proteins may have a causal role in gefitinib resistance and could be the targets in gefitinib-sensitizing effect. Furthermore, since all three validated proteins are enzymes, their activities could be evaluated in future study.

Some differential proteins, such as CAT, TXNRD1, HMOX1, and NQO1, are involved in oxidative stress, while some others, such as SCD and FABP4, are important in fatty acid metabolism. Therefore, we speculated that oxidative stress and fatty acid metabolism are the most affected processes in the sensitizing effect of CTS.

Tumor cells establish an altered redox balance with both increased levels of ROS (reactive oxygen species) and elevated levels of antioxidant proteins than its normal counterparts, making them particularly sensitive to oxidative insults (Moloney and Cotter, 2018). This suggests manipulation of ROS levels to be a promising anticancer strategy. Many studies have confirmed that increasing ROS levels drive oxidative stress-induced cancer cell death. Notably, CTS has been found to induce ROS-dependent cell death in gastric and colon cancer cells (Liu et al., 2017; Xu et al., 2017). These provide evidence that CTS performs its antitumor effect partly by affecting redox homeostasis.

CAT, a heme protein mainly in the peroxisomes of most cells, converts H_2_O_2_ into H_2_O and O_2_ in the absence of any cofactors (Reczek and Chandel, 2017). Cancer tissues are reported to have an altered expression level of CAT. Interestingly, CAT was found downregulated in human lung cancer (Ho et al., 2001; Yoo et al., 2008), suggesting that lung cancer cells are sensitive to oxidative stress. Treatment downregulating CAT has been shown efficacious to sensitize breast cancer cells to pro-oxidant therapy (Glorieux and Calderon, 2018). Therefore, CTS may sensitize lung cancer cells to gefitinib by inhibiting CAT.

HMOX1, an enzyme that degrades heme to carbon monoxide, ferrous iron, and biliverdin (Dunn et al., 2014), is highly inducible by stresses and plays a major role in protection against oxidative injuries. Elevated HMOX1 expression is found in a variety of tumors, supporting tumor cell survival, promoting proliferation and angiogenesis, as well as resisting apoptosis (Was et al., 2010). HMOX1 inhibitor suppresses thyroid tumor growth (Yang et al., 2018) and potentiates metformin efficacy in prostate cancer cells (Raffaele et al., 2019). Furthermore, inhibition of HMOX1 can also enhance cancer immunotherapy (Schillingmann et al., 2019). Collectively, inhibition of HMOX1 is a feasible anticancer approach, which could account for the activity of CTS in lung cancer.

Fatty acid metabolic reprogramming in cancer has received increasing notice for their crucial roles as structural membrane components, energy sources, and secondary messengers (Koundouros and Poulogiannis, 2020).

SCD is the enzyme that converts saturated fatty acids to Δ9-monounsaturated fatty acids, implicated in a variety of cancers. Increased expression of SCD is correlated with cancer aggressiveness and poor outcomes in patients (Tracz-Gaszewska and Dobrzyn, 2019). Many SCD inhibitors have been developed and tested preclinically. Blockade of SCD leads to reduced content of unsaturated fatty acids and suppression of NF-κB signaling, thereby restraining ovarian cancer stem cells (Li et al., 2017). Combinatory use of SCD inhibitor reverts resistance of lung cancer stem cells to cisplatin and enhances sensitivity of hepatic cancer cells to sorafenib (Ma et al., 2017; Pisanu et al., 2017).

Fatty acid-binding proteins (FABPs) are a family of highly conserved lipid chaperone molecules with varied functions. One of its members, FABP4, is reported to be overexpressed in cancers, such as prostate and ovarian cancers (Uehara et al., 2014 ; Gharpure et al., 2018), but it is found to suppress cell proliferation in hepatocellular carcinomas and endometrial cancers (Zhong et al., 2018; Wu et al., 2021). The role of FABP4 in cancer is still unclear. FABP4 has been found upregulated by CTS in the tissue sample of our study, and whether CTS enhances gefitinib efficacy through FABP4 remains to be explored.

Many studies have shown that CTS affects redox homeostasis. CTS is reported to induce ROS production, which entails autophagic cell death in A549 lung cancer cells (Hao et al., 2016). It is consistent with our observation that CTS sensitizes H1975 cells to oxidative insults by downregulating the detoxifying enzymes CAT and HMOX1. In our previous study, CTS was found to reverse cisplatin resistance in human lung carcinoma A549 cells through downregulating the Nrf2 pathway (Xia et al., 2015). Since *HMOX1* is a target gene of Nrf2, a transcription factor responsive to oxidative stress (Bai et al., 2016), it is possible that CTS suppresses HMOX1 expression by downregulating Nrf2. Interestingly, CTS is also found to ameliorate inflammation and oxidative stress by activating the Nrf2-HMOX1 pathway in nontumorous contexts (Wang et al., 2018; Zhou et al., 2019), implying a dual role of CTS in oxidative stress response. However, the dependency of resistant lung cancer on the Nrf2-HMOX1 pathway and CTS effect on this pathway remain to be explored.

CTS is found with a regulatory role in lipid metabolism. Several studies identified that CTS counters diabetes and obesity (Kim et al., 2007), and also attenuates hepatic steatosis (Nagappan et al., 2019) by activation of AMP-activated protein kinase (AMPK), a suppressor of lipogenesis (Kikuchi and Tsukamoto, 2020). We have found that CTS inhibits SCD, involving fatty acid metabolism. Concerning the relationship between SCD and AMPK, a study showed that inhibition of SCD1 in cancer cells promoted the activation of AMPK and the subsequent reduction of glucose-mediated lipogenesis (Scaglia et al., 2009). In addition, an integrated proteomic and metabolomic research on CTS in treating acne showed that CTS remedies abnormalities in unsaturated fatty acid synthetic enzymes and metabolites (Zhu et al., 2021). Therefore, it is possible that CTS modulates lipid and fatty acid metabolism in resistant cancer cells by regulating the SCD and AMPK pathways.

There are some limitations in our study. First, we studied the combination effect of CTS and gefitinib only in H1975 cell line, and whether CTS has a similar effect in other lung cancer cells remains to be further determined. Second, the underlying mechanisms of drug combination were not very clear in this study, and further in-depth research will be carried out to make the result more convincing.

In conclusion, our study offers an insight into the mechanism of CTS in enhancing the sensitivity of H1975 lung cancer cell to gefitinib. Potential target proteins have been identified by proteomics with the expression of three selected proteins validated. Bioinformatics analysis revealed the sensitizing effect of CTS on Gef therapy is associated with oxidative stress and fatty acid metabolism. These findings have clinical implications that combinatory use of CTS may be useful to treat gefitinib- resistant lung cancer.

## Data Availability

The data presented in the study are deposited in the PRIDE repository, accession number PXD031533.
