# Supplementary material for: Comparative Proteomics Analysis Reveals the Reversal Effect of Cryptotanshinone on Gefitinib-Resistant Cells in Epidermal Growth Factor Receptor-Mutant Lung Cancer
Source: Front Pharmacol. 2022 Mar 10;13:837055. doi: 10.3389/fphar.2022.837055 (PMC8965640; doi:10.3389/fphar.2022.837055)
Supplement: Supplementary file 1 [file Table1.DOCX]

| Table 1. Quantitative results of protein in H1975 cell | | | |
| --- | --- | --- | --- |
| Accession | Protein Name | Abundance  Ratio | P-Value |
| Q07283 | Trichohyalin (TCHH) | 0.01 | 5.37E-16 |
| Q70J99 | unc-13 homolog D (UNC13D) | 0.01 | 5.37E-16 |
| Q9P253 | VPS18, CORVET/HOPS core subunit (VPS18) | 0.01 | 5.37E-16 |
| P31323 | protein kinase c AMP-dependent type II regulatory subunit beta (PRKAR2B) | 0.026 | 5.37E-16 |
| O43688 | phospholipid phosphatase 2 (PLPP2) | 0.11 | 5.37E-16 |
| P15559 | NAD(P)H quinone dehydrogenase 1(NQO1) | 0.216 | 5.37E-16 |
| P05204 | high mobility group nucleosomal binding domain  2(HMGN2) | 0.242 | 5.37E-16 |
| P53985 | solute carrier family 16 member 1(SLC16A1) | 0.276 | 7.07E-10 |
| Q9NY27 | protein phosphatase 4 regulatory subunit 2(PPP4R2) | 0.298 | 5.3E-09 |
| Q53EL6 | programmed cell death 4 (neoplastic transformation inhibitor) (PDCD4) | 0.331 | 2.08E-06 |
| P00167 | cytochrome b5 type A(CYB5A) | 0.346 | 1.15E-14 |
| Q15651 | high mobility group nucleosomal binding domain  3(HMGN3) | 0.352 | 6.85E-06 |
| O95833 | chloride intracellular channel 3(CLIC3) | 0.377 | 2.28E-05 |
| P04040 | Catalase (CAT) | 0.389 | 5.49E-07 |
| P15941 | mucin 1, cell surface associated (MUC1) | 0.4 | 1.26E-10 |
| Q9C0J8 | WD repeat domain 33(WDR33) | 0.4 | 0.003055 |
| P37268 | farnesyl-diphosphate farnesyltransferase 1(FDFT1) | 0.421 | 6.47E-08 |
| Q96NE9 | FERM domain containing 6(FRMD6) | 0.427 | 0.00591 |
| P16104 | H2A histone family member X(H2AFX) | 0.436 | 1.58E-07 |

| Q9UPQ0 | LIM and calponin homology domains 1(LIMCH1) | 0.44 | 0.003757 |
| --- | --- | --- | --- |
| P26358 | DNA methyltransferase 1(DNMT1) | 0.447 | 0.002787 |
| Q15004 | KIAA0101(KIAA0101) | 0.461 | 0.00444 |
| P36405 | ADP ribosylation factor like GTPase 3(ARL3) | 0.469 | 0.002361 |
| Q14192 | four and a half LIM domains 2(FHL2) | 0.476 | 0.000749 |
| Q9H1E3 | nuclear casein kinase and cyclin dependent kinase substrate 1(NUCKS1) | 0.483 | 7E-06 |
| Q99643 | succinate dehydrogenase complex subunit C(SDHC) | 0.484 | 6.35E-05 |
| Q15800 | methylsterol monooxygenase 1(MSMO1) | 0.489 | 0.000916 |
| P24844 | myosin light chain 9(MYL9) | 0.494 | 8.31E-07 |
| O14545 | TRAF-type zinc finger domain containing  1(TRAFD1) | 0.495 | 0.006114 |
| Q14956 | glycoprotein nmb(GPNMB) | 0.505 | 0.000572 |
| Q03169 | TNF alpha induced protein 2(TNFAIP2) | 0.51 | 6.23E-09 |
| Q13268 | dehydrogenase/reductase 2(DHRS2) | 0.517 | 1.74E-09 |
| Q86TG7 | paternally expressed 10(PEG10) | 0.519 | 0.00551 |
| P50895 | basal cell adhesion molecule (Lutheran blood group) (BCAM) | 0.523 | 5.31E-05 |
| P52943 | cysteine rich protein 2(CRIP2) | 0.525 | 2.86E-05 |
| P43353 | aldehyde dehydrogenase 3 family member  B1(ALDH3B1) | 0.533 | 0.003276 |
| Q68CZ2 | tensin 3(TNS3) | 0.537 | 0.030209 |
| Q9BV57 | acireductone dioxygenase 1(ADI1) | 0.539 | 2.98E-06 |
| Q9Y2S6 | translation machinery associated 7 homolog (TMA7) | 0.541 | 0.035213 |
| Q10589 | bone marrow stromal cell antigen 2(BST2) | 0.545 | 0.00059 |
| P25325 | mercaptopyruvate sulfurtransferase(MPST) | 0.552 | 0.005094 |
| P62328 | thymosin beta 4, X-linked (TMSB4X) | 0.56 | 1.06E-06 |
| P09601 | heme oxygenase 1(HMOX1) | 0.561 | 0.00076 |

| Q01628 | interferon induced transmembrane protein  3(IFITM3) | 0.561 | 0.000488 |
| --- | --- | --- | --- |
| Q9Y613 | formin homology 2 domain containing 1(FH0D1) | 0.577 | 0.001408 |
| P10644 | protein kinase c AMP-dependent type I regulatory  subunit alpha (PRKAR1A) | 0.586 | 0.00067 |
| P21912 | succinate dehydrogenase complex iron sulfur  subunit B(SDHB) | 0.59 | 9.27E-05 |
| O14521 | succinate dehydrogenase complex subunit  D(SDHD) | 0.601 | 0.006348 |
| P11388 | topoisomerase (DNA) II alpha(T0P2A) | 0.607 | 0.034558 |
| P00403 | cytochrome c oxidase subunit II(C0X2) | 0.63 | 0.000235 |
| 000767 | stearoyl-CoA desaturase (SCD) | 0.632 | 0.018763 |
| Q13938 | Calcyphosine (CAPS) | 0.633 | 0.00045 |
| P11310 | acyl-CoA dehydrogenase, C-4 to C-12 straight chain (ACADM) | 0.635 | 0.025384 |
| Q01581 | 3-hydroxy-3-methylglutaryl-CoA synthase  1(HMGCS1) | 0.635 | 0.005825 |
| Q9Y2V2 | calcium regulated heat stable protein 1(CARHSP1) | 0.648 | 0.009395 |
| Q02880 | topoisomerase (DNA) II beta(T0P2B) | 0.661 | 0.018744 |
| 043633 | charged multivesicular body protein 2A(CHMP2A) | 0.664 | 0.024555 |
| Q8WX93 | palladin, cytoskeletal associated protein (PALLD) | 0.669 | 0.042021 |
| P08962 | CD63 molecule (CD63) | 1.521 | 0.042021 |
| Q92597 | N-myc downstream regulated 1(NDRG1) | 1.524 | 0.039772 |
| Q15758 | solute carrier family 1 member 5(SLC1A5) | 1.537 | 0.030406 |
| P41250 | glycyl-tRNA synthetase (GARS) | 1.55 | 0.001011 |
| Q9Y617 | phosphoserine aminotransferase 1(PSAT1) | 1.563 | 0.000726 |

methylenetetrahydrofolate dehydrogenase (NADP+

| P13995 | dependent) 2, methenyltetrahydrofolate | 1.582 | 0.026342 |
| --- | --- | --- | --- |
|  | cyclohydrolase(MTHFD2) |  |  |
| Q9BZQ8 | family with sequence similarity 129 member  A(FAM129A) | 1.599 | 0.014242 |
| Q9HCU5 | prolactin regulatory element binding(PREB) | 1.599 | 0.039365 |
| P78330 | phosphoserine phosphatase(PSPH) | 1.606 | 0.000501 |
| Q9UH17 | apolipoprotein B mRNA editing enzyme catalytic subunit 3B(APOBEC3B) | 1.626 | 0.023534 |
| P84243 | H3 histone family member 3A(H3F3A) | 1.628 | 0.010091 |
| P35527 | keratin 9(KRT9) | 1.632 | 0.032699 |
| P02042 | hemoglobin subunit delta(HBD) | 1.635 | 0.001854 |
| P02771 | alpha fetoprotein(AFP) | 1.651 | 0.014345 |
| O95994 | anterior gradient 2, protein disulphide isomerase family member(AGR2) | 1.668 | 0.003041 |
| P04080 | cystatin B(CSTB) | 1.68 | 3.07E-05 |
| P04264 | keratin 1(KRT1) | 1.68 | 0.000114 |
| P02765 | alpha 2-HS glycoprotein(AHSG) | 1.681 | 2.97E-05 |
| Q13751 | laminin subunit beta 3(LAMB3) | 1.689 | 0.009261 |
| A6NCE7 | microtubule associated protein 1 light chain 3 beta  2(MAP1LC3B2) | 1.701 | 0.002644 |
| Q16831 | uridine phosphorylase 1(UPP1) | 1.703 | 1.57E-05 |
| P52789 | hexokinase 2(HK2) | 1.713 | 1.14E-05 |
| Q16881 | thioredoxin reductase 1(TXNRD1) | 1.732 | 7.04E-06 |
| P13498 | cytochrome b-245 alpha chain(CYBA) | 1.741 | 0.001582 |
| P15151 | poliovirus receptor(PVR) | 1.749 | 3.55E-05 |
| Q8N183 | NADH:ubiquinone oxidoreductase complex assembly factor 2(NDUFAF2) | 1.753 | 0.003224 |

| Q06210 | glutamine--fructose-6-phosphate transaminase  1(GFPT1) | 1.762 | 4.98E-06 |
| --- | --- | --- | --- |
| P04114 | apolipoprotein B(APOB) | 1.816 | 0.001756 |
| Q9BYN0 | sulfiredoxin 1(SRXN1) | 1.908 | 0.000182 |
| Q9NUU7 | DEAD-box helicase 19A(DDX19A) | 1.93 | 0.032237 |
| P17030 | zinc finger protein 25(ZNF25) | 1.942 | 0.030406 |
| P13645 | keratin 10(KRT10) | 2.008 | 1.15E-05 |
| Q9BTE3 | minichromosome maintenance complex binding protein(MCMBP) | 2.015 | 0.004085 |
| P08243 | asparagine synthetase (glutamine-  hydrolyzing)(ASNS) | 2.018 | 9.62E-10 |
| Q9BTC0 | death inducer-obliterator 1(DIDO1) | 2.196 | 0.020449 |
| Q96BW5 | phosphotriesterase related(PTER) | 2.208 | 3.81E-08 |
| Q96EV8 | dystrobrevin binding protein 1(DTNBP1) | 2.338 | 0.010238 |
| Q6IN84 | mitochondrial rRNA methyltransferase 1(MRM1) | 2.348 | 0.002491 |
| P35908 | keratin 2(KRT2) | 2.755 | 2.3E-10 |
| P35354 | prostaglandin-endoperoxide synthase 2(PTGS2) | 2.843 | 7.54E-07 |
| 060930 | ribonuclease H1(RNASEH1) | 3.199 | 2.58E-05 |
| Q13907 | isopentenyl-diphosphate delta isomerase 1(IDI1) | 7.763 | 5.37E-16 |
| O95831 | apoptosis inducing factor, mitochondria associated  1(AIFM1) | 100 | 5.37E-16 |
| P10253 | glucosidase alpha, acid(GAA) | 100 | 5.37E-16 |
| P48637 | glutathione synthetase(GSS) | 100 | 5.37E-16 |
| P49590 | histidyl-tRNA synthetase 2, mitochondrial(HARS2) | 100 | 5.37E-16 |
| Q03405 | plasminogen activator, urokinase receptor(PLAUR) | 100 | 5.37E-16 |
| Q06787 | fragile X mental retardation 1(FMR1) | 100 | 5.37E-16 |
| Q13232 | NME/NM23 nucleoside diphosphate kinase  3(NME3) | 100 | 5.37E-16 |

| Q15274 | quinolinate phosphoribosyltransferase(QPRT) | 100 | 5.37E-16 |
| --- | --- | --- | --- |
| Q5T8P6 | RNA binding motif protein 26(RBM26) | 100 | 5.37E-16 |
| Q86YZ3 | hornerin(HRNR) | 100 | 5.37E-16 |
| Q8TD19 | NIMA related kinase 9(NEK9) | 100 | 5.37E-16 |
| Q8WW59 | SPRY domain containing 4(SPRYD4) | 100 | 5.37E-16 |
| Q96HJ9 | formation of mitochondrial complex V assembly  factor 1 homolog(FMC1) | 100 | 5.37E-16 |
| Q9NRF9 | DNA polymerase epsilon 3, accessory subunit(POLE3) | 100 | 5.37E-16 |
| Q9UKX2 | myosin heavy chain 2(MYH2) | 100 | 5.37E-16 |

Table 2. Quantitative results of protein in tumor tissues

| Accession | Protein Name | Abundance  Ratio | P-Value |
| --- | --- | --- | --- |
| O95716 | RAB3D, member RAS oncogene family(RAB3D) | 0.01 | 3.65E-16 |
| O95721 | synaptosome associated protein 29(SNAP29) | 0.01 | 3.65E-16 |
| O95999 | B-cell CLL/lymphoma 10(BCL10) | 0.01 | 3.65E-16 |
| P08575 | protein tyrosine phosphatase, receptor type  C(PTPRC) | 0.01 | 3.65E-16 |
| P09493 | tropomyosin 1 (alpha)(TPM1) | 0.01 | 3.65E-16 |
| P55212 | caspase 6(CASP6) | 0.01 | 3.65E-16 |
| P62253 | ubiquitin conjugating enzyme E2 G1(UBE2G1) | 0.01 | 3.65E-16 |
| Q14197 | mitochondrial ribosomal protein L58(MRPL58) | 0.01 | 3.65E-16 |
| Q29960 | major histocompatibility complex, class I, C(HLA-  C) | 0.01 | 3.65E-16 |
| Q5VZ46 | KIAA1614(KIAA1614) | 0.01 | 3.65E-16 |
| Q6UB35 | methylenetetrahydrofolate dehydrogenase (NADP+ | 0.01 | 3.65E-16 |

dependent) 1 -like(MTHFDlL)

| Q8N0Y7 | phosphoglycerate mutase family member  4(PGAM4) | 0.01 | 3.65E-16 |
| --- | --- | --- | --- |
| Q92959 | solute carrier organic anion transporter family  member 2A1(SLCO2A1) | 0.01 | 3.65E-16 |
| Q96MG7 | NSE3 homolog, SMC5-SMC6 complex  component(NSMCE3) | 0.01 | 3.65E-16 |
| Q99959 | plakophilin 2(PKP2) | 0.01 | 3.65E-16 |
| Q9H583 | HEAT repeat containing 1(HEATR1) | 0.01 | 3.65E-16 |
| Q9NP61 | ADP ribosylation factor GTPase activating protein  3(ARFGAP3) | 0.01 | 3.65E-16 |
| Q9NY27 | protein phosphatase 4 regulatory subunit  2(PPP4R2) | 0.01 | 3.65E-16 |
| Q9UMY1 | nucleolar protein 7(NOL7) | 0.01 | 3.65E-16 |
| Q9Y5J9 | translocase of inner mitochondrial membrane 8 homolog B(TIMM8B) | 0.01 | 3.65E-16 |
| Q06787 | fragile X mental retardation 1(FMR1) | 0.037 | 3.65E-16 |
| Q30167 | major histocompatibility complex, class II, DR beta  1(HLA-DRB1)  SWI/SNF related, matrix associated, actin | 0.079 | 3.65E-16 |
| P51532 | dependent regulator of chromatin, subfamily a, | 0.196 | 1.29E-09 |
|  | member 4(SMARCA4) |  |  |
| P06280 | galactosidase alpha(GLA) | 0.294 | 7.53E-10 |
| P46013 | marker of proliferation Ki-67(MKI67) | 0.345 | 3.29E-05 |
| Q9BUQ8 | DEAD-box helicase 23(DDX23) | 0.36 | 1.68E-06 |
| Q14694 | ubiquitin specific peptidase 10(USP10) | 0.361 | 1.15E-09 |
| Q96RP9 | G elongation factor mitochondrial 1(GFM1) | 0.362 | 3.32E-09 |
| Q12882 | dihydropyrimidine dehydrogenase(DPYD) | 0.376 | 0.000788 |

| Q04206 | RELA proto-oncogene, NF-kB subunit(RELA) | 0.393 | 9.86E-07 |
| --- | --- | --- | --- |
| Q86YZ3 | hornerin(HRNR) | 0.406 | 5.03E-05 |
| P35908 | keratin 2(KRT2) | 0.429 | 0.002486 |
| Q9ULU4 | zinc finger MYND-type containing 8(ZMYND8) | 0.477 | 0.014632 |
| P13645 | keratin 10(KRT10) | 0.478 | 3.65E-16 |
| Q71DI3 | histone cluster 2 H3 family member c(HIST2H3C) | 0.55 | 3.65E-16 |
| P84243 | H3 histone family member 3A(H3F3A) | 0.57 | 4.14E-11 |
| Q8NFJ5 | G protein-coupled receptor class C group 5 member  A(GPRC5A) | 0.585 | 0.005981 |
| O14493 | claudin 4(CLDN4) | 0.615 | 0.020687 |
| Q9NX14 | NADH:ubiquinone oxidoreductase subunit  B11(NDUFB11) | 0.626 | 0.000109 |
| Q969E2 | secretory carrier membrane protein 4(SCAMP4) | 0.642 | 0.003321 |
| Q16850 | cytochrome P450 family 51 subfamily A member  1(CYP51A1) | 0.661 | 0.007568 |
| Q3T8J9 | gon-4 like(GON4L) | 0.661 | 0.001044 |
| O14773 | tripeptidyl peptidase 1(TPP1) | 0.668 | 0.003026 |
| P06732 | creatine kinase, M-type(CKM) | 1.517 | 8.06E-15 |
| P01614 | immunoglobulin kappa variable 2-40(IGKV2-40) | 1.531 | 0.003928 |
| P41226 | ubiquitin like modifier activating enzyme 7(UBA7) | 1.547 | 0.001086 |
| P00918 | carbonic anhydrase 2(CA2) | 1.557 | 5.92E-05 |
| P31146 | coronin 1A(CORO1A) | 1.557 | 5.32E-13 |
| O14983 | ATPase sarcoplasmic/endoplasmic reticulum Ca2+ transporting 1(ATP2A1) | 1.574 | 5.04E-09 |
| P48163 | malic enzyme 1(ME1) | 1.575 | 0.000406 |
| P61956 | small ubiquitin-like modifier 2(SUMO2) | 1.589 | 0.000364 |
| Q15323 | keratin 31(KRT31) | 1.592 | 6.06E-12 |
| Q9BX10 | GTP binding protein 2(GTPBP2) | 1.608 | 4.48E-08 |

| P15104 | glutamate-ammonia ligase(GLUL) | 1.627 | 1.28E-06 |
| --- | --- | --- | --- |
| P18084 | integrin subunit beta 5(ITGB5) | 1.629 | 0.020281 |
| P06753 | tropomyosin 3(TPM3) | 1.67 | 0.005879 |
| P69905 | hemoglobin subunit alpha 1(HBA1) | 1.686 | 3.65E-16 |
| P12109 | collagen type VI alpha 1 chain(COL6A1) | 1.691 | 0.004549 |
| O95563 | mitochondrial pyruvate carrier 2(MPC2) | 1.693 | 0.02944 |
| Q9ULC5 | acyl-CoA synthetase long-chain family member  5(ACSL5) | 1.702 | 0.000759 |
| Q8N3F8 | MICAL like 1(MICALL1) | 1.712 | 3.65E-16 |
| P02042 | hemoglobin subunit delta(HBD) | 1.746 | 3.65E-16 |
| P43155 | carnitine O-acetyltransferase(CRAT) | 1.756 | 0.001895 |
| P05164 | myeloperoxidase(MPO) | 1.759 | 3.65E-16 |
| P17661 | desmin(DES) | 1.762 | 3.65E-16 |
| P48788 | troponin I2, fast skeletal type(TNNI2) | 1.792 | 0.010952 |
| P51888 | proline and arginine rich end leucine rich repeat protein(PRELP) | 1.793 | 0.003725 |
| Q9HCU5 | prolactin regulatory element binding(PREB) | 1.831 | 0.020906 |
| P07451 | carbonic anhydrase 3(CA3) | 1.913 | 3.65E-16 |
| P02788 | lactotransferrin(LTF) | 1.917 | 9.25E-09 |
| P02730 | solute carrier family 4 member 1 (Diego blood group)(SLC4A1) | 1.951 | 2.44E-06 |
| P23193 | transcription elongation factor A1(TCEA1) | 1.952 | 0.006955 |
| Q6YN16 | hydroxysteroid dehydrogenase like 2(HSDL2) | 1.998 | 0.003026 |
| P15090 | fatty acid binding protein 4(FABP4) | 2.025 | 3.65E-16 |
| O00193 | chromosome 11 open reading frame 58(C11orf58) | 2.029 | 0.007635 |
| Q8TEQ6 | gem nuclear organelle associated protein  5(GEMIN5) | 2.074 | 3.65E-16 |
| P78385 | keratin 83(KRT83) | 2.086 | 0.001266 |

| P98082 | DAB2, clathrin adaptor protein(DAB2) | 2.126 | 0.020201 |
| --- | --- | --- | --- |
| P15586 | glucosamine (N-acetyl)-6-sulfatase(GNS) | 2.149 | 0.000101 |
| H7BZ55 | ciliary rootlet coiled-coil, rootletin family member  2(CROCC2) | 2.251 | 5.39E-12 |
| Q16270 | insulin like growth factor binding protein  7(IGFBP7) | 2.272 | 0.000416 |
| P50750 | cyclin dependent kinase 9(CDK9) | 2.353 | 0.015642 |
| Q9GZY8 | mitochondrial fission factor(MFF) | 2.469 | 0.008866 |
| Q8NDH3 | aminopeptidase-like 1(NPEPL1) | 2.472 | 0.00028 |
| P35609 | actinin alpha 2(ACTN2) | 2.544 | 2.57E-07 |
| P68133 | actin, alpha 1, skeletal muscle(ACTA1) | 2.56 | 6.61E-05 |
| Q8N142 | adenylosuccinate synthase like 1(ADSSL1) | 2.64 | 5.03E-05 |
| P13807 | glycogen synthase 1(GYS1) | 2.684 | 3.65E-16 |
| P25189 | myelin protein zero(MPZ) | 2.853 | 3.65E-16 |
| P21695 | glycerol-3-phosphate dehydrogenase 1(GPD1) | 2.866 | 3.65E-16 |
| P78318 | immunoglobulin (CD79A) binding protein  1(IGBP1) | 2.891 | 0.000851 |
| Q6P1M0 | solute carrier family 27 member 4(SLC27A4) | 2.958 | 7.97E-05 |
| P11217 | phosphorylase, glycogen, muscle(PYGM) | 3.016 | 3.31E-07 |
| P28330 | acyl-CoA dehydrogenase, long chain(ACADL) | 3.068 | 1.91E-13 |
| Q6ZRV2 | family with sequence similarity 83 member  H(FAM83H) | 3.259 | 6.11E-08 |
| Q3LXA3 | triokinase and FMN cyclase(TKFC) | 3.662 | 1.32E-09 |
| Q9NR33 | DNA polymerase epsilon 4, accessory subunit(POLE4) | 3.98 | 7.63E-09 |
| P56378 | chromosome 14 open reading frame 2(C14orf2) | 4.039 | 3.65E-16 |
| Q96AC1 | fermitin family member 2(FERMT2) | 4.693 | 4.13E-12 |
| Q9NSB4 | keratin 82(KRT82) | 5.785 | 3.65E-16 |

| Q13363 | C-terminal binding protein 1(CTBP1) | 8.201 | 3.65E-16 |
| --- | --- | --- | --- |
| Q8TCS8 | p olyrib onucl eoti de nucleotidyltransferase  1(PNPT1) | 8.762 | 3.65E-16 |
| O14896 | interferon regulatory factor 6(IRF6) | 15.533 | 3.65E-16 |
| Q99996 | A-kinase anchoring protein 9(AKAP9) | 23.369 | 3.65E-16 |
| Q7Z794 | keratin 77(KRT77) | 98.276 | 3.65E-16 |
| A0A0B4J  2A2 | peptidylprolyl isomerase A like 4C(PPIAL4C) | 100 | 3.65E-16 |
| O43719 | HIV-1 Tat specific factor 1(HTATSF1) | 100 | 3.65E-16 |
| O76013 | keratin 36(KRT36) | 100 | 3.65E-16 |
| O76031 | caseinolytic mitochondrial matrix peptidase  chaperone subunit(CLPX) | 100 | 3.65E-16 |
| P00390 | glutathione-disulfide reductase(GSR) | 100 | 3.65E-16 |
| P06865 | hexosaminidase subunit alpha(HEXA) | 100 | 3.65E-16 |
| P30622 | CAP-Gly domain containing linker protein  1(CLIP1) | 100 | 3.65E-16 |
| P49459 | ubiquitin conjugating enzyme E2 A(UBE2A) | 100 | 3.65E-16 |
| P52757 | chimerin 2(CHN2) | 100 | 3.65E-16 |
| P98194 | ATPase secretory pathway Ca2+ transporting  1(ATP2C1) | 100 | 3.65E-16 |
| Q58FG0 | heat shock protein 90 alpha family class A member  5, pseudogene(HSP90AA5P) | 100 | 3.65E-16 |
| Q7L5D6 | golgi to ER traffic protein 4(GET4) | 100 | 3.65E-16 |
| Q8IXM2 | chromosome 17 open reading frame 49(C17orf49) | 100 | 3.65E-16 |
| Q8NBX0 | saccharopine dehydrogenase (putative)(SCCPDH) | 100 | 3.65E-16 |
| Q8WXA9 | splicing regulatory glutamic acid and lysine rich protein 1(SREK1) | 100 | 3.65E-16 |
| Q92575 | UBX domain protein 4(UBXN4) | 100 | 3.65E-16 |

| Q93009 | ubiquitin specific peptidase 7(USP7) | 100 | 3.65E-16 |
| --- | --- | --- | --- |
| Q9BYR9 | keratin associated protein 2-4(KRTAP2-4) | 100 | 3.65E-16 |
| Q9H4I3 | TraB domain containing(TRABD) | 100 | 3.65E-16 |
| Q9HCD5 | nuclear receptor coactivator 5(NCOA5) | 100 | 3.65E-16 |
| Q9NRY2 | INTS3 and NABP interacting protein(INIP) | 100 | 3.65E-16 |
| Q9P013 | CWC15 spliceosome associated protein  homolog(CWC15) | 100 | 3.65E-16 |
| Q9Y5P4 | collagen type IV alpha 3 binding  protein(COL4A3BP) | 100 | 3.65E-16 |
| Q58FG0 | heat shock protein 90 alpha family class A member  5, pseudogene(HSP90AA5P) | 100 | 3.65E-16 |
| Q7L5D6 | golgi to ER traffic protein 4(GET4) | 100 | 3.65E-16 |
| Q8IXM2 | chromosome 17 open reading frame 49(C17orf49) | 100 | 3.65E-16 |
| Q8NBX0 | saccharopine dehydrogenase (putative)(SCCPDH) | 100 | 3.65E-16 |
| Q8WXA9 | splicing regulatory glutamic acid and lysine rich protein 1(SREK1) | 100 | 3.65E-16 |
| Q92575 | UBX domain protein 4(UBXN4) | 100 | 3.65E-16 |
| Q93009 | ubiquitin specific peptidase 7(USP7) | 100 | 3.65E-16 |
| Q9BYR9 | keratin associated protein 2-4(KRTAP2-4) | 100 | 3.65E-16 |
| Q9H4I3 | TraB domain containing(TRABD) | 100 | 3.65E-16 |
| Q9HCD5 | nuclear receptor coactivator 5(NCOA5) | 100 | 3.65E-16 |
| Q9NRY2 | INTS3 and NABP interacting protein(INIP) | 100 | 3.65E-16 |
| Q9P013 | CWC15 spliceosome associated protein  homolog(CWC15) | 100 | 3.65E-16 |
| Q9Y5P4 | collagen type IV alpha 3 binding  protein(COL4A3BP) | 100 | 3.65E-16 |
